# Validation of Dynamic Deuterium Metabolic Imaging (DMI) for the Measurement of Cerebral Metabolic Rates of Glucose in Rat

**DOI:** 10.1002/nbm.70194

**Published:** 2025-12-10

**Authors:** Claudius S. Mathy, Monique A. Thomas, Graeme F. Mason, Robin A. de Graaf, Henk M. De Feyter

**Affiliations:** ^1^ Department of Radiology and Biomedical Imaging, Magnetic Resonance Research Center Yale University New Haven Connecticut USA; ^2^ Institute of Physical and Theoretical Chemistry University of Bonn Bonn Germany; ^3^ Institute of Radiology, University Hospital Erlangen, Friedrich‐Alexander‐Universität Erlangen‐Nürnberg (FAU) Erlangen Germany; ^4^ Department of Biomedical Engineering Yale University New Haven Connecticut USA

**Keywords:** deuterium metabolic imaging (DMI), metabolic modeling, MRS

## Abstract

Deuterium metabolic imaging (DMI) is an innovative technique in which ^2^H magnetic resonance spectroscopic imaging (MRSI) is utilized to determine the metabolic activity of administered ^2^H‐labeled substrates. As such it can be viewed as the ^2^H counterpart to more traditional ^13^C labeling methods that can be considered the gold standard for metabolic mapping in vivo. To ensure reliable findings from dynamic ^2^H MRSI experiments about absolute metabolic flux rates after administration of a ^2^H‐labeled substrate it is essential to take into account ^2^H‐specific aspects, namely ^2^H label losses and kinetic isotopy effects (KIEs). Here, a modified version of a ^13^C‐based metabolic model for glucose metabolism in rat brain was developed to address these ^2^H‐related effects, tested for ^2^H MRSI data acquired during infusion of [6,6'‐^2^H_2_]‐glucose, and validated by comparison with indirect ^1^H‐[^13^C] MRSI data acquired during infusion of [1‐^13^C]‐glucose. The flux rates for glucose consumption (CMR_gl_ = 0.57 ± 0.08 μmol/min/g) and the TCA cycle (V_tca_ = 1.24 ± 0.14 μmol/min/g) derived from the ^2^H MRSI data and using the updated metabolic model were in excellent agreement with the estimates based on ^13^C data (CMR_gl_ = 0.59 ± 0.14 μmol/min/g and V_tca_ = 1.24 ± 0.32 μmol/min/g). The successful validation of dynamic ^2^H MRSI for absolute flux rate determination forms the basis for future quantitative study of metabolic disorders in vivo.

## Graphical abstract

Deuterium metabolic imaging (DMI) is a novel metabolic imaging technique where 2H MRSI is combined with the administration of 2H‐labeled substrates. Analysis of dynamic DMI is complicated by 2H label losses and kinetic isotope effects. Here, we modified a 13C‐based metabolic model to account for these effects in rat brain and validated DMI flux rates of glucose consumption and the TCA cycle by comparison with indirect 1H‐[13C] MRSI. Flux rates determined by both techniques were in excellent agreement.

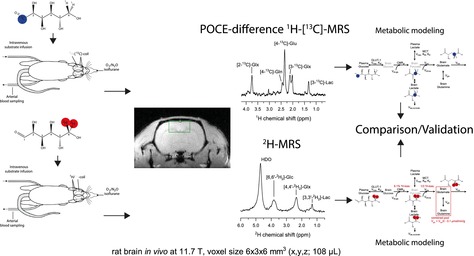

**Abbreviations**
CMR_gl_
: flux rate for glucose consumption
DMI: deuterium metabolic imaging
FE: fractional enrichment
Glc: glucose
Gln: glutamine
Glu: glutamate
Glx: combined pool of Glu and Gln
KIE: kinetic isotope effect
Lac: lactate
MRSI: magnetic resonance spectroscopic imaging
POCE: proton‐observed carbon‐edited
RF: radio frequency
V_tca_
: flux rate for the TCA cycle

## 1 Introduction

Deuterium metabolic imaging (DMI) combines ^2^H MR spectroscopic imaging (MRSI) with the administration of deuterium‐labeled substrates to map active metabolism in vivo [1, 2]. So far, clinically oriented applications of DMI have focused predominantly on mapping ^2^H‐labeling during isotopic steady state, the time period during which ^2^H MR signals of ^2^H‐enriched Lac and Glx are close to constant [1, 3, 4, 5, 6]. In such a scenario, DMI data are acquired following the administration of deuterated glucose and a waiting period, in analogy with clinically used ^18^F‐deoxyglucose positron emission tomography. The labeling detected during steady state is the result of the active metabolism of the labeled glucose, but does not allow for the derivation of metabolic rates. Instead, when DMI data are acquired dynamically, as a time series that captures the full kinetics of the ^2^H‐labeling in downstream metabolites, absolute quantification of metabolic rates is feasible [2, 7, 8, 9]. This approach is equivalent to the use of ^13^C‐labeled substrates and the detection of ^13^C‐labeling kinetics with direct ^13^C or indirect ^1^H‐[^13^C] MRS methods [10, 11]. However, DMI has several advantages, including simplicity and robustness, and higher sensitivity than direct ^13^C MRS because the short T_1_ of ^2^H allows for increased averaging for a given time period [1, 12]. This higher effective sensitivity offers the opportunity to detect ^2^H in MRSI mode at a relevant spatial resolution. In contrast, detection of active metabolism of ^13^C‐labeled substrates using MRSI is only realistic when using ^1^H‐based, indirect ^13^C detection such as proton‐observed carbon‐edited (POCE) MRSI. However, POCE is technically more complex because of the need for water suppression, high‐quality localization and lipid suppression, high magnetic field homogeneity, and ideally also includes heteronuclear decoupling [10].

A frequent application of ^13^C MRS in vivo is the study of brain metabolism using glucose ^13^C‐labeled in the 1st and/or 6th carbon position [11, 13, 14, 15, 16]. Most human brain studies use [1‐^13^C]‐glucose because of the high cost of [1,6‐^13^C_2_]‐glucose. If instead DMI is used, [6,6'‐^2^H_2_]‐glucose is the preferred labeled glucose because of its two deuterons and relative affordability.

The spectral resolution of ^2^H MR spectra is lower than that of ^1^H and ^13^C MR spectra, and results in the overlap of the individual glutamate (Glu) and glutamine (Gln) peaks even at available ultra‐high field scanners [1, 2]. Therefore, unlike ^13^C or ^1^H‐[^13^C] MRS methods, DMI does not allow for studying Glu‐Gln neurotransmitter cycling, which requires the quantification of labeling in these separate pools. Yet, using the combined pool of Glu + Gln (Glx), dynamic DMI can be a relatively simple, robust and considerably cost‐effective technique to noninvasively study brain glucose metabolism in vivo, in a spatially resolved way.

Absolute quantification of the cerebral metabolic rate based on isotope tracer studies, for example involving ^2^H or ^13^C, requires fitting kinetic data to a mathematical metabolic model. The model used for ^2^H‐glucose is similar to those used for ^13^C‐glucose‐based studies but is restricted to a single neural compartment because of the need to use a combined Glx pool, instead of modeling neuronal and astroglial fluxes separately. In addition, ^2^H‐based metabolic models should take into account the fractional ^2^H label loss [17, 18]. Another difference between ^2^H and ^13^C‐based metabolic modeling is that the brain ^2^H‐glucose levels can be used directly as the input function, whereas POCE‐based modeling typically calculates brain ^13^C‐glucose levels from the measured blood glucose dynamics [11].

While theoretically sound, the novelty of using deuterated glucose with dynamic DMI for quantitative studies of cerebral glucose metabolism warrants validation of this approach. We used both [1‐^13^C]‐glucose and [6,6'‐^2^H_2_]‐glucose in two separate groups of rats and collected data on labeling kinetics from the same brain region with localized POCE and ^2^H MRS, respectively. As such, we validated the use of [6,6’‐^2^H_2_]‐glucose to study brain glucose metabolism with dynamic DMI, and provided a practical metabolic model that is available to the research community. Dynamic DMI combined with metabolic modeling can be used to study glucose metabolism in healthy and diseased brains with high spatial resolution compared to direct ^13^C approaches. Regional differences in glucose metabolism are known or suspected in many of the diseases and conditions that affect the brain. A spatially resolved, dynamic DMI approach is therefore expected to increase our understanding of how and which areas of the brain are impacted by such diseases, as well as by how potential therapies can affect glucose metabolism.

## 2 Methods

### 2.1 MR Systems

In vivo experiments were performed on an 11.7 T magnet (Magnex Scientific Ltd., Yarnton, UK) interfaced to an Avance III HD spectrometer running on ParaVision 6 (Bruker, Billerica, MA, USA) equipped with 9.0 cm diameter gradients and 0.5 kW radiofrequency (RF) amplifiers for ^1^H/^2^H/^13^C transmission.

For ^2^H MRS, ^2^H RF transmission and reception were performed with a two‐turn 20 mm × 15 mm elliptical surface coil tuned to ^2^H (76.7 MHz) integrated within two larger orthogonal 20‐mm diameter ^1^H coils driven in quadrature and tuned to ^1^H (499.8 MHz) that served for ^1^H‐MRI acquisition and shimming (see Figure S1) [1, 19].

For ^1^H‐[^13^C] MRS, a similar coil design was utilized where ^1^H and X coils are interchanged, with a single‐turn 14‐mm diameter coil tuned to ^1^H (499.8 MHz) and two 20‐mm diameter coils tuned to ^13^C for ^1^H‐[^13^C] editing and ^13^C broadband decoupling (125.7 MHz) [10].

High‐resolution NMR scans were acquired using a 500‐MHz NMR spectrometer (Avance III 500, Bruker) and were run on TopSpin 3.2 using 5‐mm probes for ^1^H and X‐nucleus acquisitions.

### 2.2 Animal Preparation/Glucose Substrate

Healthy Fischer344 rats (*n* = 8 each for ^2^H MRS and ^1^H‐[^13^C] MRS) were anesthetized with a mixture of 30/70% O_2_/N_2_O and 1.5% isoflurane via a nose cone. Body temperature was maintained at ~37°C using a heating water pad, and breathing monitored by a pressure sensor (MouseOx, Starr Life Sciences Corp., PA, USA). Catheters were placed in the femoral artery for blood sampling and blood pressure monitoring, and in the femoral vein for glucose infusion while acquiring in vivo MRS data. For infusion, either [6,6‐^2^H_2_]‐glucose or [1‐^13^C]‐glucose (Cambridge Isotopes Laboratories Inc., Tewksbury, MA, USA) were dissolved in water to a concentration of 1 M and a bolus continuous infusion protocol was utilized resulting in 1.95 g/kg body weight of [6,6‐^2^H_2_]‐glucose infused within a 120 min study [1]. Blood samples collected at 0, 5, 9, 20, 40, 60, 90, and 120 min were spun down and the plasma supernatant stored at −80°C until further analysis. Plasma samples were treated as previously described [1]. Briefly, after centrifugation and methanol precipitation, the samples were resuspended in phosphate buffer (100 mM), with D_2_O (10%), sodium formate as chemical shift reference and imidazole as an internal concentration reference. All animal procedures were approved by the Yale University Institutional Animal Care and Use Committee.

### 2.3 MR Signal Acquisition

For anatomical localization proton‐density weighted MR images were acquired with a gradient‐echo sequence (^2^H MRS:, TR/TE = 100/3.3 ms, 30°, field of view (FOV) of 27.0 × 27.0 mm^2^ and 6 slices of 1.0 mm thickness; ^1^H‐[^13^C] MRS TR/TE = 3000/2 ms, 45°, FOV of 25.5 × 25.5 mm^2^ and 6 slices of 1.0 mm thickness, see Table S2).

Second‐order spherical harmonical shimming after B_0_‐mapping resulted for ^2^H MRS in a HDO linewidth of 14–27 Hz across 8 × 5 × 8 or 7 × 4 × 7 mm^3^ volumes.

To achieve sharp boundaries, 2D (^2^H MRS) and 3D (POCE) volumes were selected, followed by 1D phase encoding. The POCE method required 3D volume selection to prevent potential signal contamination from lipid signal originating from the scalp. For ^2^H MRS in rodents this is not necessary because of the low levels of lipids and low natural abundance of ^2^H (< 0.015%). For ^2^H MRS a 2D column of 6 × 6 mm in the dorsal‐ventral direction was selected, after which 1D phase‐encoding provided localization along the third dimension. ^2^H MRS used a spin‐echo sequence [TR/TE = 800/8 ms, spectral width (SW) = 5 kHz, and 9 phase encoding steps over 27 mm]. Signal excitation was achieved with a slice‐selective 90° Shinnar‐Le‐Roux‐(SLR) RF pulse (1 ms), followed by two 180° adiabatic pulses (2 ms). 64 averages led to a total acquisition time of 7 min 40 s.

^1^H‐[^13^C] MRS data were obtained using a POCE MRSI sequence that included VAPOR water suppression, 3D LASER volume selection and adiabatic ^13^C‐decoupling [10, 20, 21, 22]. Signal excitation was achieved by a 0.5 ms adiabatic half passage pulse (tanh/tan RF/frequency modulation, *R* = bandwidth × pulse length = 100) [23] followed by LASER localization executed with six 0.8 ms adiabatic full passage (AFP) pulses (sech/tanh modulation, *R* = 20) [22]. Inversion on the ^13^C channel was achieved by a 1 ms AFP pulse (sech4 modulation, *R* = 25) [24]. All adiabatic pulses were executed at a power level well above the minimum threshold to reach adiabaticity. An additional phase‐encoding gradient (ventral—dorsal y‐orientation) was applied directly after the ^13^C inversion pulse and lead to a spatial resolution of 6 × 1.5 × 6 mm^3^. 4 averages (TR = 4000 ms, TE = 21 ms + 7.8 ms for heteronuclear scalar‐coupling evolution, SW = 10 kHz and 17 phase‐encoding increments) resulted in a total acquisition time of 9 min 04 s. A macromolecular baseline was measured in one animal utilizing T_1_ relaxation time differences between macromolecules and metabolites (TR = 5000 ms, TI_1_ = 1950 ms, TI_2_ = 550 ms). For the spectral quantification, two 1.5 mm thick POCE volumes were combined to match the single, 3 mm thick ^2^H MRS volume. This approach allowed for better spectral quality in the POCE spectra due to more homogenous B_0_ in the individual, smaller voxels. The sensitivity penalty from combining voxels after acquisition was not a concern because the intrinsic higher SNR of ^1^H‐[^13^C] MR spectra compared to ^2^H MR spectra.

High‐resolution ^1^H NMR data of blood plasma were acquired with a pulse‐acquire sequence (TR = 18 s, NA = 256) utilizing water pre‐saturation and adiabatic ^2^H decoupling. For samples of the ^1^H‐[^13^C] MRS study, ^2^H WALTZ decoupling was omitted, all other parameters were identical.

### 2.4 MR Signal Processing

In vivo ^2^H and ^1^H‐[^13^C] MRS data were phase‐shifted in the y‐direction to assure that the upper edge of the 6 × 3 × 6 mm^3^ voxel of selected brain tissue coincides with the upper edge of the rat brain in ventral‐dorsal orientation (see Figure 2A for an example for voxel localization). The phase shift was achieved by recalculating the position of the phase encoding steps according to the so‐called shift theorem (de Graaf, 2019, in vivo NMR spectroscopy). ^2^H and ^1^H‐[^13^C] MRS data were further pre‐processed by zero‐filling the free‐induction decays to 4096 points, Fourier‐transformed in the spatial and spectral domain, and phase‐corrected with in‐house written scripts in MATLAB R2019b (MathWorks, Natick, MA, USA). Spectral quantification was performed with an in‐house written algorithm for linear combination of model spectra. Spectra used for display purposes in figures presenting dynamic time courses (Figures 3B and 4B) were zero‐filled to 32,768 points and line‐broadened by a 2.0 Hz Gaussian, in all other cases zero‐filled to 4096 points without line‐broadening.

In vivo ^2^H MR spectra are modeled as a superposition of a linear baseline and a basis set of deuterated metabolites: HDO, [6,6'‐^2^H_2_]‐glucose, [4,4'‐^2^H_2_]‐Glx, and [3,3'‐^2^H_2_]‐Lac. The basis set used a combination of four peaks with Lorentzian line shape for glucose, the rest of the metabolites and HDO were modeled as singlets. All metabolite concentrations (in mM) were based on the pre‐infusion, natural abundance water signal, equal to 10.12 mM (determined assuming 55.5 M water concentration, 80% water content in brain tissue and ^2^H natural abundance of 0.0115%) [1]. Brain metabolite concentrations were converted to μmol/g assuming rat brain density of 1.1 g/mL^2^.

The in vivo ^1^H‐[^13^C] MR difference spectra were also modeled as the sum of a linear baseline and a basis set of individual ^13^C‐labeled metabolites: alanine (Ala), aspartate (Asp), GABA, Glu, Gln, Lac, and N‐acetyl aspartate (NAA). The associated total ^1^H MRS spectra were modeled similarly, with addition of creatine (Cr), phosphocreatine (PCr), NAA‐Glu (NAAG) and the acquired macromolecular baseline (MM). The total metabolite concentrations were calculated by using the total creatine (sum of PCr and Cr, tCr) as internal standard of 10 μmol/g. Due to large variation in the values of Lac obtained from fitting the total spectra, the total Lac concentration was not based on these spectra but included as a fitted parameter within the metabolic model, described below. To illustrate the impact on metabolic modeling, the analyses were additionally carried out with Lac concentration values obtained from the total spectra.

High‐resolution spectra of blood plasma were evaluated with a home‐written graphical user interface within MATLAB. The total Glc concentration for ^2^H MRS experiments was calculated by peak integration and referencing the *α*‐H6 signal of glucose to the H4/H5‐signal of the concentration standard imidazole in the ^1^H NMR spectra. FE of [6,6'‐^2^H_2_]‐ glucose was evaluated using the *β*H6 signal, according to FE = 100 × (1—S_*β*H6_/S_*β*glucose_), whereby S_*β*glucose_ is the total single‐proton *β*‐glucose signal obtained from multiplying the glucose *α*‐H1 signal by the *β*‐to‐*α* anomeric ratio, 0.63/0.37. Total and ^2^H‐labeled concentrations of Lac were determined by spectral fitting of unlabeled, single‐labeled and double‐labeled Lac, and a first‐order baseline, as described previously [17]. For ^1^H‐[^13^] MRS, the glucose and Lac FEs were calculated based on the *α*‐[1‐^13^C]‐H1, and the H3 Lac signal respectively, and their ^13^C satellites. The total concentration of glucose and Lac was calculated using the imidazole internal concentration standard.

### 2.5 Metabolic Modeling

**FIGURE 1:**
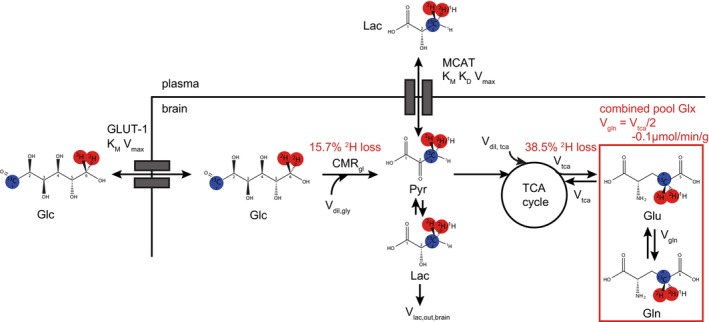
Metabolic model. Glucose (Glc) is transported across the blood–brain barrier and into neurons by transporters characterized by Michaelis–Menten kinetics with K_M_ and V_max_, metabolized by CMR_gl_ to pyruvate that is in isotopic equilibrium with Lac. The Lac pool can also have a contribution from Lac formed outside of the brain that can enter via monocarboxylic acid transporters characterized by Michaelis–Menten kinetic parameters K_M_ and V_max_, and a saturable component K_D_. Lac/pyruvate is metabolized after entering the TCA cycle (V_tca_) to *α*‐KG that is in rapid exchange with Glu. The Glu/Gln cycle is characterized by V_gln_. ^2^H and ^13^C label positions are indicated in red or blue (for simplicity both label positions are marked within one molecule). Unlabeled substrate in‐ and outflows are V_dil_,_gly_ = V_lac_,_out_,_brain_, and V_dil_,_tca_. Modifications for ^2^H compared to ^13^C MRS are indicated in red including correction factors for ^2^H label loss during glycolysis and the TCA cycle. The combined Glu/Gln pool is connected by V_gln_ that is in a linear relationship with oxidative brain metabolism characterized by V_tca_.

To compare the rate estimates of glucose consumption (CMR_gl_) and TCA cycle (V_TCA_) between ^2^H MRS and ^1^H‐[^13^C] MRS, a simplified two‐compartment model (plasma and brain) was used with specific adaptations for ^2^H MRS data [25]. The model was implemented in CWave for kinetic fitting, and which generated the associated mass and isotope balance equations [26]. A schematic of the metabolic fluxes is shown in Figure 1, and the associated mass and isotope balance equations are provided in Table S1.

In brief, plasma time courses of glucose and Lac serve as driver functions, with the reversible transport of both moles across the blood–brain barrier being modeled using Michaelis–Menten kinetics. The transport parameters Michaelis–Menten constant K_M_, and maximum velocity V_max_, were fitted using the plasma and brain levels of deuterated glucose, with previously reported estimates as starting values [27]. The ^2^H‐MRS‐based average values of K_M_ and V_max_ were used as constants during the modeling of the ^1^H‐[^13^C] MRS data. Transport parameters for both ^2^H and ^13^C‐labeled Lac were based on previous reports [28].

Time courses of the ^2^H or ^13^C‐labeled brain metabolites Lac and Glx are the target functions to be fitted based on the metabolic model. CMR_gl_ reflects the flux of brain glucose to pyruvate that is in isotopic equilibrium with the brain Lac pool [25]. Deuterium label loss affects the isotope flow but not the mass flow in the metabolic model. Therefore the isotope flux equation was modified to reflect the 15.7% ^2^H label loss at [3,3'‐^2^H_2_]‐Lac [17]. Other fluxes affecting the deuterated Lac pool embedded in the model include plasma lactate in/outflow, and contribution of brain glycogen.

The ^2^H or ^13^C label enters the TCA cycle via pyruvate dehydrogenase (V_pdh_) and labels carbon position four in the *α*‐ketoglutarate (*α*‐KG) pool. Together with unlabeled inflows V_dil,gly_, the sum of these fluxes constitutes V_tca_. Assuming that *α*‐ketoglutarate is in rapid exchange with Glu (V_x_ > > V_tca_), the rate of Glu labeling reflects the rate of V_tca_. Glu is further metabolized to Gln via the glutamate/glutamine cycle (V_gln_) or within the TCA cycle at the rate of V_tca_. Because ^2^H‐labeled Glu and Gln cannot be separated due to spectral overlap, Glu and Gln were combined in Glx with a previously determined relationship between V_tca_ and V_gln_ [29]. Previous work determined ^2^H label loss occurring between the Lac and the Glx pool at 37.9% for Glu and 41.5% of Gln [17]. A resulting 38.5% concentration‐weighted average (from ^13^C data) label loss of Glx was taken into account by including this loss in the isotope flow equation (Table S1). Even though ^1^H‐[^13^C] MRS can distinguish between Glu and Gln, ^13^C data were also modeled with a combined Glx pool to minimize model differences and enhance overall consistency.

Previous studies showed minor ^2^H KIE on metabolic flux rates within the rat brain, therefore deuterium‐based CMR_gl_ was multiplied by the k_H_/k_D_ ratio = 1.042 for lactate and V_tca_ by the k_H_/k_D_ ratio of Glu = 1.035 [17].

The metabolic model consists of a set of coupled differential equations for isotope and mass balance. For mass balance equations, it was assumed that the pool sizes remain constant over time with the exception of glucose. The metabolic flux rates CMR_gl_ and V_tca_, and transport parameters of Glc (^2^H only) were iterated to yield the best fit for the time courses of [6,6'‐^2^H_2_]‐ glucose, [4,4'‐^2^H_2_]‐Glx and [3,3'‐^2^H_2_]‐Lac for ^2^H, and [4‐^13^C]‐Glx and [3‐^13^C]‐Lac for ^13^C as target functions, and the plasma glucose and Lac time courses as driver functions using the Levenberg–Marquardt‐algorithm in CWave.

## 3 Results

**FIGURE 2:**
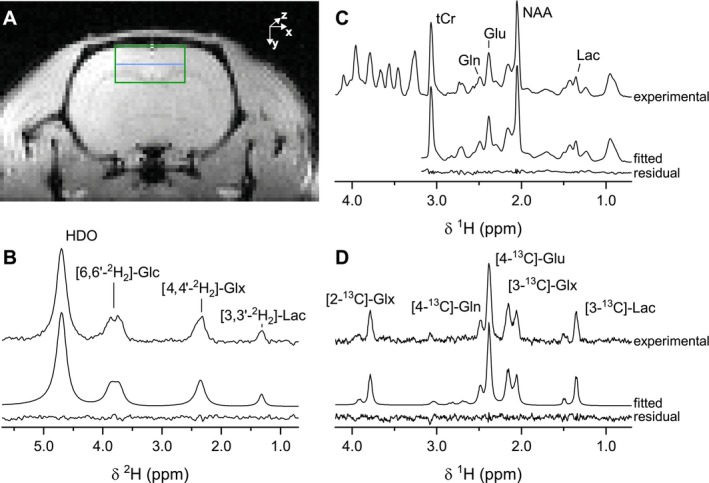
^2^H and ^1^H‐[^13^C] MRS in vivo. (A) Axial proton‐density weighted image with the 6 × 3 × 6 mm^3^ voxel marked in green. Additional blue line indicates the two ^1^H‐[^13^C] MRS volumes that were combined. B, C and D, Experimental ^2^H MR (B), total ^1^H‐[^13^C] MR (C) and difference ^1^H‐[^13^C] MR (D) spectra of the rat brain combined with the best spectral fit, and residual.

Figure 2A shows the position of the 6 × 3 × 6 mm^3^ voxel on a transversal proton‐density weighted MR image. ^1^H‐[^13^C] MRS spectra were combined to yield the same voxel volume at similar spatial localization as for ^2^H MRS. Figure 2B–D shows examples of ^2^H, total ^1^H and edited ^1^H‐[^13^C] MR spectra, best fit, and fitting residue, acquired between 90 and 120 min after the start of a ^2^H‐ or ^13^C‐labeled glucose infusion. These examples illustrate the spectral fit quality for glucose (^2^H only), the metabolites Glu/Gln labeled at carbon position 4, Glx (^2^H only) and Lac labeled at position 3, which represent the metabolites used to evaluate brain glucose metabolism. Total metabolite concentrations are shown in Figure S2.

**FIGURE 3:**
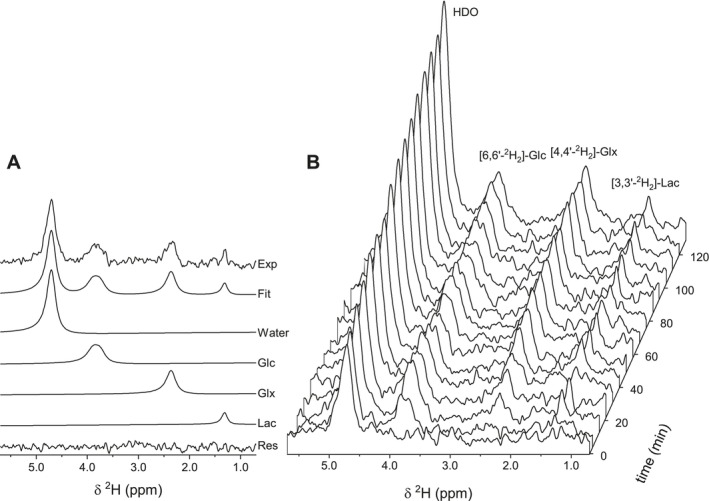
Dynamic ^2^H MRS of rat brain in vivo. (A) Experimental ^2^H MR spectrum acquired at 49 min together with the best fit, contributions from water, Glc, Glx and Lac. (B) Dynamic ^2^H MR spectra acquired during 120 min of intravenous [6,6'‐^2^H_2_]‐Glc with a temporal resolution of 7.4 min.

**FIGURE 4:**
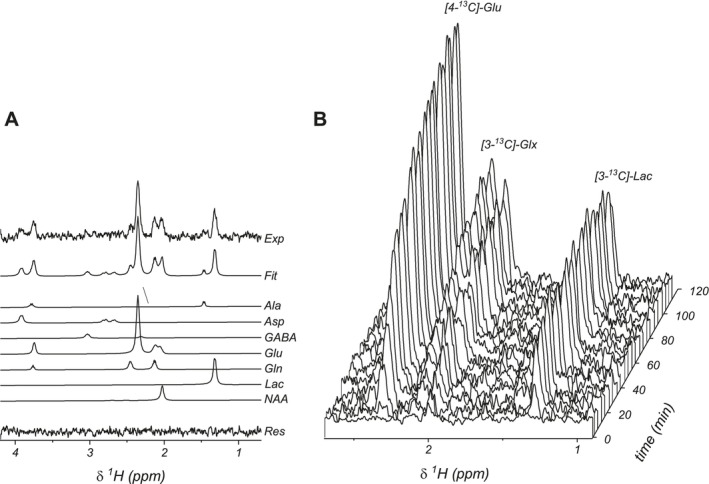
Dynamic ^1^H‐[^13^C] MRS of rat brain in vivo. (A) Experimental difference ^1^H‐[^13^C] MR spectrum acquired at 50 min together with the best fit, contributions from Ala, Asp, GABA, Glu, Gln, Lac, and NAA. (B) Dynamic ^1^H‐[^13^C] MR spectra acquired during 120 min of intravenous [1‐^13^C]‐Glc with a temporal resolution of 4.5 min.

Figures 3A and 4A show the spectral fitting of ^2^H and ^1^H‐[^13^C] spectra. Dynamically acquired ^2^H MRS spectra display a linear increase in the water signal, reflecting whole‐body glucose metabolism (Figure 3B). Brain [6,6'‐^2^H_2_]‐glucose shows rapid accumulation with subsequent stable levels, followed by the appearance of [3,3'‐^2^H_2_]‐Lac and the [4,4'‐^2^H_2_]‐Glx signal. ^1^H‐[^13^C] MRS showed a similar time course of metabolite label enrichment as for ^2^H MRS, with appearance of [3‐^13^C]‐Lac, immediately followed by [4‐^13^C]‐Glu and [4‐^13^C]‐Gln (Figure 4B). The higher spectral resolution of ^1^H MRS compared to ^2^H MRS allowed for Glu and Gln to be detected with limited overlap. [3‐^13^C]‐Glx and [2‐^13^C]‐Glx are formed in subsequent turns of the TCA cycle. The equivalent ^2^H‐labeled counterparts are not detected due to ^2^H‐label loss downstream of [4,4'‐^2^H_2_]‐*α*‐KG in the TCA‐cycle. Glucose could not be detected with ^1^H‐[^13^C] MRS due to water suppression affecting the H1 glucose resonances.

**FIGURE 5:**
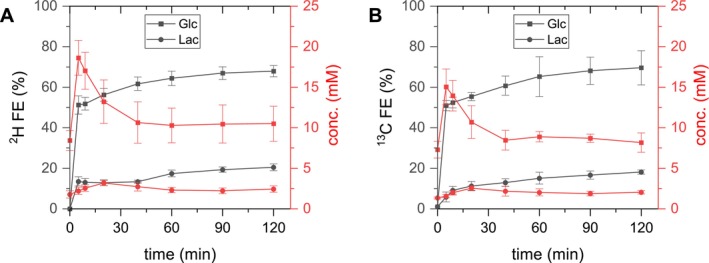
Plasma glucose. Plasma Glc and Lac concentrations and FE during infusion of either [6,6'‐^2^H_2_]‐Glc (A) or [1‐^13^C]‐Glc (B).

Time courses of average concentrations and FEs of plasma [6,6'‐^2^H_2_]‐ and [1‐^13^C]‐glucose are displayed in Figure 5A,B. Glucose concentrations increased from 8.4 ± 1.2 and 7.3 ± 1.0 mM (^2^H vs. ^13^C *p* = 0.064) pre‐infusion to 18.6 ± 2.2 and 15.0 ± 2.2 mM within 5 min (^2^H vs. ^13^C *p* = 0.005), then decreased to 10.6 ± 2.6 and 8.4 ± 1.3 mM at 40 min (^2^H vs. ^13^C *p* = 0.818) and remained stable until the end of the infusion for ^2^H, and ^13^C, respectively. High FEs of 51 ± 5 and 51 ± 3% (^2^H vs. ^13^C *p* = 0.908) were achieved within 5 min, slowly increased until 40 min and were at 68 ± 3 and 70 ± 8% (^2^H vs. ^13^C *p* = 0.637) at 120 min, for ^2^H, and ^13^C, respectively. As the FEs and the concentrations of Lac were relatively high in the plasma with concentrations of 2.4 ± 0.4 and 2.0 ± 0.2 mM (^2^H vs. ^13^C *p* = 0.022) and FEs of 20 ± 2 and 18 ± 1% (^2^H vs. ^13^C p = 0.005) at 120 min for ^2^H, and ^13^C, respectively, plasma Lac was included as an input function in the metabolic model.

**FIGURE 6:**
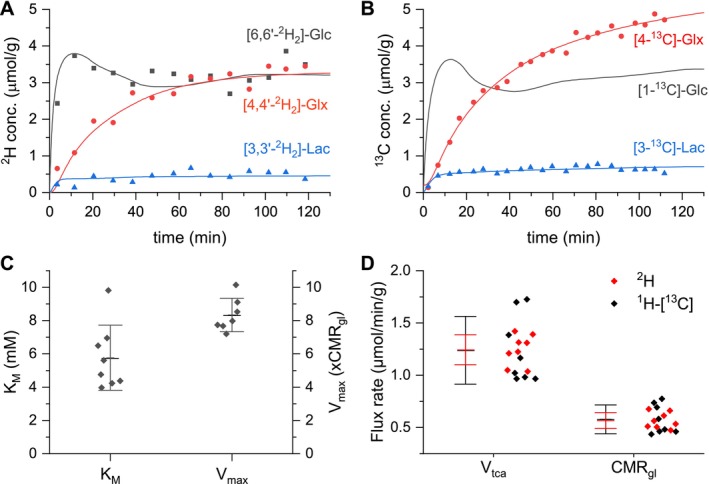
Metabolic modeling. ^2^H and ^13^C label turnover for flux rate determination in rat brain in vivo. Examples of experimental data together with the best fit (solid lines) obtained from metabolic modeling for [6,6'‐^2^H_2_]‐Glc (A) and [1‐^13^C]‐Glc (B) infusion experiments. (C) Transport characteristics determined within metabolic modeling of ^2^H MRS characterized by Michaelis–Menten kinetic parameters K_M_ and V_max_. (D) Metabolic flux rates of glucose consumption and TCA cycle, CMR_gl_, and V_tca_ for ^2^H MRS (red) and ^1^H‐[^13^C] MRS (black).

Figure 6A,B shows examples of metabolic fitting of ^2^H and ^13^C‐labeled metabolite time courses. Since [6,6'‐^2^H_2_]‐glucose was detectable in the brain, the parameters for glucose transport over the blood–brain barrier could be determined by best fit within the metabolic modeling, of resulting in K_M_ = 5.8 ± 2.0 mM and V_max_/CMR_gl_ = 8.5 ± 1.0 (Figure 6C). These values were applied to the ^13^C data to derive the brain glucose concentration based on the plasma glucose levels.

End point ^2^H‐labeled brain metabolite concentrations (averaged over 30 min 40 s) were 3.6 ± 0.6 μmol/g for [6,6'‐^2^H_2_]‐ glucose, 0.5 ± 0.1 μmol/g for [3,3'‐^2^H_2_]‐Lac and 3.0 ± 0.3 μmol/g for [4,4'‐^2^H_2_]‐Glx, whereas ^2^H FE was 26.9 ± 3.3% for Lac, and 14.7 ± 1.4% for Glx. Average end point ^13^C‐labeled metabolite concentrations (averaged over 27 min 12 s) were 0.7 ± 0.5 μmol/g for [3‐^13^C]‐Lac (*p* = 0.22 vs. ^2^H) and 5.6 ± 1.0 μmol/g for [4‐^13^C]‐Glx (4.7 ± 0.9 mmol/g for Glu and 0.9 ± 0.2 mol/g for Gln; *p* < 0.001 vs. ^2^H), with corresponding ^13^C FEs of 28 ± 6% for Lac (*p* = 0.71 vs. ^2^H) and 27 ± 5% for Glx (32 ± 5% for Glu and 16 ± 6% for Gln; p < 0.001 vs. ^2^H).

The lower concentrations and FE of ^2^H‐labeled Glx compared to ^13^C‐labeled Glx is a result of ^2^H label loss [10]. After correcting for the deuterium label loss and an overall correction factor for the KIEs as described in section 2.5, metabolic modeling of ^2^H MRS data led to an average CMR_gl_ of 0.57 ± 0.08 μmol/min/g and V_tca_ of 1.24 ± 0.14 μmol/min/g. These values are in excellent agreement with the values determined from modeling the ^13^C MRS data, resulting in estimates for CMR_gl_ of 0.59 ± 0.14 μmol/min/g (^2^H vs. ^13^C: *p* = 0.846) and V_tca_ of 1.24 ± 0.32 μmol/min/g (Figure 6 D; ^2^H vs. ^13^C: *p* = 0.968). Changing the total lactate pool size within the metabolic modeling to experimentally determined values from total ^1^H‐[^13^C] MRS led only to relatively small changes (mostly *< 15*%) in V_tca_ and CMR_gl_. For ^1^H‐[^13^C] MRS, V_tca_ changed from 1.24 ± 0.32 μmol/min/g to 1.38 ± 0.24 μmol/min/g and CMR_gl_ from 0.59 ± 0.14 μmol/min/g to 0.58 ± 0.10 μmol/min/g. For ^2^H MRS, V_tca_ changed from 1.24 ± 0.14 μmol/min/g to 1.40 ± 0.07 μmol/min/g and CMR_gl_ from 0.57 ± 0.08 μmol/min/g to 0.70 ± 0.04 μmol/min/g (see Figure S3).

## 4 Discussion

The approach of combining deuterium MRSI with the administration of a ^2^H‐labeled substrate of interest is increasingly gaining traction as a robust method for metabolic imaging in vivo. This method, DMI, is conceptually identical to the use of ^13^C‐labeled substrates and detection with direct or indirect ^13^C MRS(I). However, using DMI for absolute quantification of metabolic fluxes requires corrections for ^2^H label loss and KIEs [4, 10]. Previously we experimentally determined the degree of the KIE and ^2^H label loss for [6,6'‐^2^H_2_]‐glucose when metabolized in the brain. In this work we applied the ^2^H label loss and KIE corrections and validated the estimates of the cerebral metabolic rate by comparison with indirect ^1^H‐[^13^C] MRS. We also described a simple mathematical model for dynamic DMI data to estimate metabolic fluxes of glucose in the brain.

The key advantage of DMI is the rapid signal averaging that allows the buildup of sufficient signal‐to‐noise to perform MRSI at a spatial resolution useful for generating metabolic maps. For this study the focus was on validating DMI‐based metabolic rate estimates, which are independent of the chosen localization technique, and apply to both single voxel and MRSI‐based data. We used 1D phase‐encoding within a 2D column with the hope that the compartments selected within the column could be analyzed individually. The top one would contain more gray matter, and the lower compartment would contain more white matter (corpus callosum). In pilot experiments it became clear that the signal‐to‐noise ratio for DMI was relatively low in the individual compartments, and therefore the spatial resolution of the phase encoding was changed to select a 3 mm deep volume. Given the higher sensitivity of the ^1^H MRS‐based POCE method, we continued with two 1.5 mm thick compartments and summed the signal afterwards. This approach benefitted the quality of the POCE spectra due to the better B0 homogeneity in the smaller volumes, and still allowed us to compare DMI with POCE, because the final volume was similar in size and position, and contained comparable amounts of white and gray matter.

To address the ^2^H label loss and the KIEs an established metabolic model for cerebral flux rate determination of ^13^C MRS data was slightly modified. Deuterium label loss was incorporated in the model equation describing the fractional enrichment evolution over time [10]. Alternatively, the individual fractional enrichment values of each metabolite could have been corrected for each measured time point. By applying the correction with one step within the metabolic model, preprocessing of the data before fitting the labeling dynamics is not needed. Using the model without any compensation for ^2^H label loss could lead to less accurate and precise flux rates for CMR_gl_ and V_tca_. Underestimating primary (and secondary) KIEs would lead to a slower flux rate estimate. Since the KIEs in the brain after infusion of [6,6'‐^2^H_2_]‐glucose were shown to be less than 5%, the impact of KIE was again corrected within the model by multiplying the flux rates of CMR_gl_ and V_tca_ with the previously determined k_H_/k_D_ [17].

The final modification to the established metabolic model was to combine the Glu and Gln metabolite pools into a single pool of Glx because the spectral resolution of ^2^H MRS is insufficient to detect the individual resonances of these metabolites. This modification was accomplished within the metabolic model by using the well‐described linear relationship between oxidative neuro‐energetic metabolism and the Glu/Gln neurotransmitter cycle [29, 30]. A combined Glx pool was also used for the ^13^C MRSI data to keep the metabolic modeling of both ^2^H and ^13^C‐based data consistent.

Due to the need for water suppression in the POCE method robust detection of the nearby H1 peaks of glucose (5.2 ppm) is compromised and not suited for quantification. As a result the input function of the plasma to brain typically relies on plasma‐based data and modeling of the glucose transport, and uses literature values for its parameters. In contrast, in the ^2^H MR spectra, the peak from [6,6'‐^2^H_2_]‐glucose (3.82 ppm) can be readily observed and quantified. We therefore used these data to derive the plasma‐to‐brain glucose transport parameters, which fell within the relatively large range of previously reported values [27, 31]. As the glucose infusion protocol and related experimental conditions were identical for the ^2^H and ^13^C experiments, we could apply the glucose transport parameters in the model to curve fit the ^13^C‐based metabolic fluxes.

The glucose infusion resulted in a small but significant amount of lactate labeling. Because plasma lactate can cross the blood‐brain barrier in both directions and function as a predominantly neuronal substrate, we included this potential flux in the metabolic model using literature values for lactate transport characteristics. An infusion protocol that raises glucose to lower levels could potentially reduce systemic lactate production.

The present work follows experiments performed to determine the degree of ^2^H label loss and KIE of [6,6'‐^2^H_2_]‐glucose (and ^2^H‐acetate) during metabolism in rat brain. We anticipate that the estimates for label loss and KIE can be applied in other organs, as long as the enzymes involved in the metabolic pathway of interest are similar as in the brain. For canonical pathways such as glycolysis this is expected to be the case. Other experimental characteristics such as age and sex could possibly affect metabolic rates through unrelated mechanisms such as perfusion, substrate and cofactor availability or redox potential but are not expected to affect ^2^H label loss, or KIE. However, ^2^H label loss is position‐specific within a molecule. As such, the ^2^H loss for, e.g., glucose labeled in the first carbon position is not necessarily comparable to the deuterons of the sixth carbon position [18].

The metabolic modeling of ^2^H and ^13^C MRS‐based data resulted in similar estimates for rates of CMR_gl_ and V_tca_, validating the use of dynamic ^2^H MRS(I) data for quantitation of cerebral metabolic rates when including corrections for deuterium label loss, and (to a much lesser extent) for KIEs. However, overall the metabolic rate estimates were relatively high compared to literature values based on seemingly similar experimental conditions. Hansen et al. found similar estimates for CMR_gl_ by autoradiography that are in excellent agreement with our results under similar anesthesia conditions with isoflurane [32]. Others reported similar results or lower rates for CMR_gl_ and V_tca_ than found in this study under similar or different anesthesia conditions (halothane or morphine) [30, 32, 33]. A study by Lu et al. was the first to demonstrate the use of ^2^H‐labeled glucose to quantify cerebral metabolism in rats, and also found lower metabolic rates [2]. We can only speculate that the 2% isoflurane used in their study resulted in a deeper level of anesthesia and subsequent lower metabolic rate. While the same anesthetic was used in the present study, the level was repeatedly adjusted to keep the animals' breathing rate between 70 and 100 breaths per minute, often resulting in isoflurane levels of less than 1.5%. The range of cerebral metabolic rate estimates found in the literature indicates how several factors can affect the measurement, including the contribution of different brain tissue types, anesthesia protocols and animal strains. This justifies our approach for a comparative study with as much as possible similar conditions to validate ^2^H‐glucose‐based estimates of cerebral metabolism. Yet, our approach to keeping experimental conditions between ^2^H and ^13^C experiments identical could not be objectively evaluated, which is a limitation of this study. We used respiratory rate as the main indicator of anesthesia depth, which in our experience reflects the level of anesthesia and systemic stability, provided core body temperature is maintained. However, a separate measurement of brain activity, such as electroencephalogram, could have been added to ensure that anesthesia depth was indeed similar between animals. Alternatively, one could also interleave ^1^H‐[^13^C] and ^2^H MRS data acquisitions within the same experiment while infusing a 50:50 mix of ^13^C and ^2^H‐labeled glucose. Though this would come at the cost of a 50% lower level of fractional enrichment for each of the respective isotopes. This would also require a triple‐tuned RF coil, but such an elegant approach has recently been shown to be feasible for detection of ^1^H, ^17^O and ^2^H MRS data [34].

Deuterium MR spectra are not as rich in information as ^1^H‐[^13^C] MR spectra. Yet, as shown here and in other studies, relevant and quantitative metabolic information can be derived from ^2^H MRS data with significantly simpler and more robust acquisition methods [2]. High‐quality POCE spectra require excellent water suppression, stable experimental conditions, and typically also broadband decoupling, while also using multiple in‐line filters on the RF chain to avoid noise injection. It can be argued that this level of complexity has hampered the widespread adoption of this technique. In contrast, ^2^H MRS(I) is increasingly and rapidly being used in both preclinical and clinical research applications, presumably because of the relatively ease of its implementation and robustness [9, 35, 36, 37, 38, 39]. In addition, metabolic modeling of labeling kinetics from ^2^H‐glucose is inherently less complex than from ^13^C‐glucose because ^2^H label is lost downstream of Glx labeling and does not label *α*‐KG in the second turn of the TCA cycle [1, 2]. This makes the fitting process simpler and more stable, and could possibly be a factor contributing to the lower inter‐animal variation in the ^2^H‐based metabolic rate estimates observed in the present study.

Deuterium labeling has also been measured using an indirect approach based on the reduction in ^1^H signal of metabolites that become deuterated (quantitative exchange label turnover, QELT) [40, 41]. The metabolic model, including the label loss correction, should be directly applicable to this type of data when using a combined Glx metabolite pool. Since at ultra‐high field the QELT method allows individual quantification of Glu and Gln, the metabolic model could be slightly modified to contain both these metabolite pools and include their respective individual ^2^H label loss corrections.

In our first DMI study in humans we used oral administration of deuterated glucose followed by a waiting period and data acquisition to detect ^2^H‐labeling at (quasi‐) steady state [1]. Since then, other groups have used oral administration and acquired time course data to measure ^2^H‐labeling kinetics and estimate metabolic rates [7, 42, 43]. In theory, the model described here can be applied to time course data collected after oral administration provided that the plasma input function is sufficiently sampled. However, the determination of V_tca_ depends heavily on the difference in time course of the blood glucose enrichment and the brain Glx enrichment. Furthermore, as previous research using ^13^C‐labeled glucose has shown, the data acquisition had to be extended up to 3 h to partly remedy the precision of the metabolic rate estimates as based on a 2‐h long intravenous infusion study.

DMI is predominantly implemented as a spectroscopic imaging method using 3D matrices that can cover the whole brain. A higher spatial resolution (larger matrices) takes more time to be acquired. Our analysis (data not shown) has indicated that for human brain V_tca_ measurements time courses of isotope labeling require a minimum time resolution of 8 min. Faster turnover rates would need to be sampled at a higher time resolution. Therefore, 3D ^2^H MRSI data acquisition needs to be implemented such that it balances the spatial resolution and number of averages to achieve a sufficient level of SNR while keeping the total acquisition time at 8 min or less.

DMI could be an alternative for other metabolic imaging methods used to study the brain, such as hyperpolarized ^13^C‐pyruvate or ^18^F‐deoxyglucose positron emission tomography ([18] FDG‐PET). The advantage of DMI includes relative simplicity and robustness, and the possibility to calculate absolute value metabolic fluxes (when combined with ^1^H MRSI for metabolite pool size measurements). While the spatial resolution is lower compared to [18] FDG‐PET, the lack of ionizing radiation can facilitate many repeated scans and possibly also studying pediatric populations.

## 5 Conclusion

In this study, dynamic ^2^H MRS combined with the infusion of [6,6'‐^2^H_2_]‐glucose has been successfully validated for determining spatially localized metabolic flux rates of glucose metabolism, CMR_gl_ and V_tca_ within the healthy rat brain, by comparison with an established method, ^1^H‐[^13^C] MRS. This was achieved by adjusting an accepted metabolic model for ^13^C‐based data that addresses ^2^H label losses and KIEs, and by using a combined Glx metabolite pool. The result is a relatively straightforward metabolic model that is available for use in the research community, and can be a tool to further develop the use of ^2^H‐based metabolic imaging applications. Follow‐up studies based on the described metabolic model could provide absolute metabolic flux rate estimates for a range of brain diseases, and can also form a basis for metabolic flux modeling in organ systems other than the brain.

## Author Contributions

C.S.M. conceptualization, formal analysis, investigation, methodology, validation, writing – original draft preparation, writing – review and editing; M.A.T. formal analysis, investigation; G.F.M. formal analysis, investigation, writing – review and editing; R.A.G. funding acquisition, conceptualization, methodology, formal analysis, investigation, writing – review and editing; H.M.D.F. funding acquisition, resources, supervision, conceptualization, formal analysis, investigation, methodology, validation, writing – original draft preparation, writing – review and editing.

## Funding

This research was funded, in part by US National Institutes of Health grants R03 CA267438, R01EB033764, R01EB025840, and R01CA288833. C.S.M. was founded by the Deutsche Forschungsgemeinschaft (DFG, German Research Foundation)—493624887 (Clinician Scientist Program NOTICE).

## Conflicts of Interest

All authors declare no conflicts of interest.

## Supporting information


**Figure S1:** Coil configuration and positioning for ^1^H‐[^13^C] MRS (top) and ^2^H MRS (bottom).

**Figure S2:** Total metabolic concentrations (pool sizes) as obtained from in vivo total ^1^H‐[^13^C] MRS under the assumption of a 10 μmol/g tCr concentration. Total Lac concentration obtained from metabolic modeling of ^1^H‐[^13^C] MRS data.

**Figure S3:** Metabolic flux rates of glucose consumption and TCA cycle, CMR_gl_ and V_tca_ for ^2^H MRS (red) and ^1^H‐[^13^C] MRS (black) when changing the total lactate pool size within the metabolic modeling to experimentally determined values for ^1^H‐[^13^C] MRS compared to Figure 6D where the total lactate pool size is iterated as a free parameter of the metabolic modeling.

**Data S1:** Supplementary equation.

**Data S2:** Supplementary information.

## Data Availability

Data generated or analyzed during the study are available from the corresponding author by reasonable request. CWave, including the metabolic models, is available by contacting co‐author G.F.M.

## References

[1] H. M. De Feyter , K. L. Behar , Z. A. Corbin , et al., “Deuterium Metabolic Imaging (DMI) for MRI‐Based 3D Mapping of Metabolism In Vivo,” Science Advances 4, no. 8 (2018): eaat7314, 10.1126/sciadv.aat7314. 30140744 PMC6105304

[2] M. Lu , X.‐H. Zhu , Y. Zhang , G. Mateescu , and W. Chen , “Quantitative Assessment of Brain Glucose Metabolic Rates Using In Vivo Deuterium Magnetic Resonance Spectroscopy,” Journal of Cerebral Blood Flow and Metabolism 37, no. 11 (2017): 3518–3530, 10.1177/0271678X17706444. 28503999 PMC5669347

[3] N. Bøgh , M. Aastrup , J. K. Mortensen , et al., “Comparison of Deuterium Metabolic Imaging With FDG PET in Alzheimer Disease,” Radiology 315, no. 1 (2025): e241808, 10.1148/radiol.241808. 40197092

[4] P. M. Adamson , K. Datta , R. Watkins , L. D. Recht , R. E. Hurd , and D. M. Spielman , “Deuterium Metabolic Imaging for 3D Mapping of Glucose Metabolism in Humans With Central Nervous System Lesions at 3T,” Magnetic Resonance in Medicine 91, no. 1 (2024): 39–50, 10.1002/mrm.29830. 37796151 PMC10841984

[5] J. D. Kaggie , A. S. Khan , T. Matys , et al., “Deuterium Metabolic Imaging and Hyperpolarized 13C‐MRI of the Normal Human Brain at Clinical Field Strength Reveals Differential Cerebral Metabolism,” NeuroImage 257 (2022): 119284, 10.1016/j.neuroimage.2022.119284. 35533826

[6] E. Serés Roig , H. M. De Feyter , T. W. Nixon , et al., “Deuterium Metabolic Imaging of the Human Brain In Vivo at 7 T,” Magnetic Resonance in Medicine 89, no. 1 (2023): 29–39. 36063499 10.1002/mrm.29439 PMC9756916

[7] L. Ruhm , N. Avdievich , T. Ziegs , et al., “Deuterium Metabolic Imaging in the Human Brain at 9.4 Tesla With High Spatial and Temporal Resolution,” NeuroImage 244 (2021): 118639, 10.1016/j.neuroimage.2021.118639. 34637905 PMC8591372

[8] S. Frese , B. Strasser , L. Hingerl , et al., “Balanced Steady‐State Free Precession Enables High‐Resolution Dynamic 3D Deuterium Metabolic Imaging of the Human Brain at 7T,” Investigative Radiology. Published online December 13 (2024), 10.1097/RLI.0000000000001196. PMC12353933 40273422

[9] F. Niess , B. Strasser , L. Hingerl , et al., “Whole‐Brain Deuterium Metabolic Imaging via Concentric Ring Trajectory Readout Enables Assessment of Regional Variations in Neuronal Glucose Metabolism,” Human Brain Mapping 45, no. 6 (2024): e26686, 10.1002/hbm.26686. 38647048 PMC11034002

[10] R. A. de Graaf , D. L. Rothman , and K. L. Behar , “State of the Art Direct 13C and Indirect 1H‐[13C] NMR Spectroscopy In Vivo. A Practical Guide,” NMR in Biomedicine 24, no. 8 (2011): 958–972, 10.1002/nbm.1761. 21919099 PMC3694136

[11] D. L. Rothman , H. M. De Feyter , R. A. de Graaf , G. F. Mason , and K. L. Behar , “13C MRS Studies of Neuroenergetics and Neurotransmitter Cycling in Humans,” NMR in Biomedicine 24, no. 8 (2011): 943–957, 10.1002/nbm.1772. 21882281 PMC3651027

[12] R. A. de Graaf , A. D. Hendriks , D. W. J. Klomp , et al., “On the Magnetic Field Dependence of Deuterium Metabolic Imaging,” NMR in Biomedicine 33, no. 3 (2020): e4235, 10.1002/nbm.4235. 31879985 PMC7141779

[13] D. L. Rothman , R. A. de Graaf , F. Hyder , G. F. Mason , K. L. Behar , and H. M. D. Feyter , “In Vivo 13C and 1H‐[13C] MRS Studies of Neuroenergetics and Neurotransmitter Cycling, Applications to Neurological and Psychiatric Disease and Brain Cancer,” NMR in Biomedicine 32, no. 10 (2019): e4172, 10.1002/nbm.4172. 31478594

[14] K. C. C. van de Ven , C. J. Tack , A. Heerschap , M. van der Graaf , and G., B. E. de , “Patients With Type 1 Diabetes Exhibit Altered Cerebral Metabolism During Hypoglycemia,” Journal of Clinical Investigation 123, no. 2 (2013): 623–629, 10.1172/JCI62742. 23298837 PMC3561817

[15] F. Boumezbeur , G. F. Mason , R. A. de Graaf , et al., “Altered Brain Mitochondrial Metabolism in Healthy Aging as Assessed by In Vivo Magnetic Resonance Spectroscopy,” Journal of Cerebral Blood Flow and Metabolism 30, no. 1 (2010): 211–221, 10.1038/jcbfm.2009.197. 19794401 PMC2949111

[16] J. Shen , K. F. Petersen , K. L. Behar , et al., “Determination of the Rate of the Glutamate/Glutamine Cycle in the Human Brain by *In Vivo* ^13^C NMR,” PNAS 96, no. 14 (1999): 8235–8240, 10.1073/pnas.96.14.8235. 10393978 PMC22218

[17] R. A. de Graaf , M. A. Thomas , K. L. Behar , and H. M. De Feyter , “Characterization of Kinetic Isotope Effects and Label Loss in Deuterium‐Based Isotopic Labeling Studies,” ACS Chemical Neuroscience 12, no. 1 (2021): 234–243. 33319987 10.1021/acschemneuro.0c00711 PMC9890388

[18] O. Ben‐Yoseph , P. B. Kingsley , and B. D. Ross , “Metabolic Loss of Deuterium From Isotopically Labeled Glucose,” Magnetic Resonance in Medicine 32, no. 3 (1994): 405–409. 7984074 10.1002/mrm.1910320317

[19] G. Adriany and R. Gruetter , “A Half‐Volume Coil for Efficient Proton Decoupling in Humans at 4 Tesla,” Journal of Magnetic Resonance 125, no. 1 (1997): 178–184, 10.1006/jmre.1997.1113. 9245377

[20] I. Tkáč , Z. Starčuk , I. Y. Choi , and R. Gruetter , “In Vivo ^1^H NMR Spectroscopy of Rat Brain at 1 ms Echo Time,” Magnetic Resonance in Medicine 41, no. 4 (1999): 649–656, 10.1002/(SICI)1522-2594(199904)41:4<649::AID-MRM2>3.0.CO;2-G. 10332839

[21] M. A. Kukurugya , S. Rosset , and D. V. Titov , “The Warburg Effect Is the Result of Faster ATP Production by Glycolysis Than Respiration,” National Academy of Sciences of the United States of America 121, no. 46 (2024): e2409509121, 10.1073/pnas.2409509121. PMC11573683 39514306

[22] M. Garwood and L. DelaBarre , “The Return of the Frequency Sweep: Designing Adiabatic Pulses for Contemporary NMR,” Journal of Magnetic Resonance 153, no. 2 (2001): 155–177, 10.1006/jmre.2001.2340. 11740891

[23] M. Garwood and Y. Ke , “Symmetric Pulses to Induce Arbitrary Flip Angles With Compensation for RF Inhomogeneity and Resonance Offsets,” Journal of Magnetic Resonance 94, no. 3 (1991): 511–525, 10.1016/0022-2364(91)90137-I.

[24] A. Tannús and M. Garwood , “Improved Performance of Frequency‐Swept Pulses Using Offset‐Independent Adiabaticity,” Journal of Magnetic Resonance Series A 120, no. 1 (1996): 133–137, 10.1006/jmra.1996.0110.

[25] G. F. Mason , D. L. Rothman , K. L. Behar , and R. G. Shulman , “NMR Determination of the TCA Cycle Rate and Alpha‐Ketoglutarate/Glutamate Exchange Rate in Rat Brain,” Journal of Cerebral Blood Flow and Metabolism 12, no. 3 (1992): 434–447, 10.1038/jcbfm.1992.61. 1349022

[26] G. F. Mason , K. Falk Petersen , R. A. de Graaf , T. Kanamatsu , T. Otsuki , and D. L. Rothman , “A Comparison of ^13^C NMR Measurements of the Rates of Glutamine Synthesis and the Tricarboxylic Acid Cycle During Oral and Intravenous Administration of [1‐^13^C]Glucose,” Brain Research Protocols 10, no. 3 (2003): 181–190, 10.1016/S1385-299X(02)00217-9. 12565689

[27] G. F. Mason , K. L. Behar , D. L. Rothman , and R. G. Shulman , “NMR Determination of Intracerebral Glucose Concentration and Transport Kinetics in Rat Brain,” Journal of Cerebral Blood Flow and Metabolism 12, no. 3 (1992): 448–455, 10.1038/jcbfm.1992.62. 1569138

[28] W. M. Pardridge and W. H. Oldendorf , “Transport of Metabolic Substrates Through the Blood‐Brain Barrier1,” Journal of Neurochemistry 28, no. 1 (1977): 5–12, 10.1111/j.1471-4159.1977.tb07702.x. 833603

[29] N. R. Sibson , A. Dhankhar , G. F. Mason , D. L. Rothman , K. L. Behar , and R. G. Shulman , “Stoichiometric Coupling of Brain Glucose Metabolism and Glutamatergic Neuronal Activity,” PNAS 95, no. 1 (1998): 316–321. 9419373 10.1073/pnas.95.1.316 PMC18211

[30] R. A. de Graaf , G. F. Mason , A. B. Patel , D. L. Rothman , and K. L. Behar , “Regional Glucose Metabolism and Glutamatergic Neurotransmission in Rat Brain In Vivo,” Proceedings of the National Academy of Sciences of the United States of America 101, no. 34 (2004): 12700–12705, 10.1073/pnas.0405065101. 15310848 PMC515118

[31] I. Y. Choi , S. P. Lee , S. G. Kim , and R. Gruetter , “In Vivo Measurements of Brain Glucose Transport Using the Reversible Michaelis–Menten Model and Simultaneous Measurements of Cerebral Blood Flow Changes During Hypoglycemia,” Journal of Cerebral Blood Flow and Metabolism 21, no. 6 (2001): 653–663, 10.1097/00004647-200106000-00003. 11488534

[32] T. D. Hansen , D. S. Warner , M. M. Todd , and L. J. Vust , “The Role of Cerebral Metabolism in Determining the Local Cerebral Blood Flow Effects of Volatile Anesthetics: Evidence for Persistent Flow‐Metabolism Coupling,” Journal of Cerebral Blood Flow and Metabolism 9, no. 3 (1989): 323–328, 10.1038/jcbfm.1989.50. 2715204

[33] F. Hyder , R. P. Kennan , I. Kida , G. F. Mason , K. L. Behar , and D. Rothman , “Dependence of Oxygen Delivery on Blood Flow in Rat Brain: A 7 Tesla Nuclear Magnetic Resonance Study,” Journal of Cerebral Blood Flow and Metabolism 20, no. 3 (2000): 485–498, 10.1097/00004647-200003000-00007. 10724113

[34] G. Zhang , P. Jenkins , W. Zhu , W. Chen , and X. H. Zhu , “Simultaneous Assessment of Cerebral Glucose and Oxygen Metabolism and Perfusion in Rats Using Interleaved Deuterium (2H) and Oxygen‐17 (^17^O) MRS,” NMR in Biomedicine 38, no. 1 (2025): e5284, 10.1002/nbm.5284. 39503302 PMC11602644

[35] F. Hesse , V. Somai , F. Kreis , F. Bulat , A. J. Wright , and K. M. Brindle , “Monitoring Tumor Cell Death in Murine Tumor Models Using Deuterium Magnetic Resonance Spectroscopy and Spectroscopic Imaging,” PNAS 118, no. 12 (2021): e2014631118, 10.1073/pnas.2014631118. 33727417 PMC8000230

[36] R. V. Simões , R. N. Henriques , B. M. Cardoso , F. F. Fernandes , T. Carvalho , and N. Shemesh , “Glucose Fluxes in Glycolytic and Oxidative Pathways Detected In Vivo by Deuterium Magnetic Resonance Spectroscopy Reflect Proliferation in Mouse Glioblastoma,” NeuroImage: Clinical 33 (2022): 102932, 10.1016/j.nicl.2021.102932. 35026626 PMC8760481

[37] C. Taglang , G. Batsios , J. Mukherjee , et al., “Deuterium Magnetic Resonance Spectroscopy Enables Noninvasive Metabolic Imaging of Tumor Burden and Response to Therapy in Low‐Grade Gliomas,” Neuro‐Oncology 24, no. 7 (2022): 1101–1112, 10.1093/neuonc/noac022. 35091751 PMC9248401

[38] D. C. Peters , S. Markovic , Q. Bao , et al., “Improving Deuterium Metabolic Imaging (DMI) Signal‐To‐Noise Ratio by Spectroscopic Multi‐Echo bSSFP: A Pancreatic Cancer Investigation,” Magnetic Resonance in Medicine 86, no. 5 (2021): 2604–2617. 34196041 10.1002/mrm.28906

[39] A. D. Hendriks , A. Veltien , I. J. Voogt , A. Heerschap , T. W. J. Scheenen , and J. J. Prompers , “Glucose Versus Fructose Metabolism in the Liver Measured With Deuterium Metabolic Imaging,” Frontiers in Physiology 14 (2023): 14, 10.3389/fphys.2023.1198578. PMC10351417 37465695

[40] L. J. Rich , P. Bagga , N. E. Wilson , et al., “ ^1^ H Magnetic Resonance Spectroscopy of ^2^ H‐to‐ ^1^ H Exchange Quantifies the Dynamics of Cellular Metabolism In Vivo,” Nature Biomedical Engineering 4 (2020): 335–342, 10.1038/s41551-019-0499-8. PMC7071956 31988460

[41] P. Bednarik , D. Goranovic , A. Svatkova , et al., “ ^1^H Magnetic Resonance Spectroscopic Imaging of Deuterated Glucose and of Neurotransmitter Metabolism at 7 T in the Human Brain,” Nature Biomedical Engineering 7 (2023): 1001–1013, 10.1038/s41551-023-01035-z. PMC10861140 37106154

[42] N. Ahmadian , M. M. Konig , S. Otto , et al., “Human Brain Deuterium Metabolic Imaging at 7 T: Impact of Different [6,6′‐^2^H_2_]Glucose Doses,” Journal of Magnetic Resonance Imaging 61, no. 3 (2025): 1170–1178, 10.1002/jmri.29532. 39058248 PMC11803682

[43] X. Li , X. H. Zhu , Y. Li , et al., “Quantitative Mapping of Key Glucose Metabolic Rates in the Human Brain Using Dynamic Deuterium Magnetic Resonance Spectroscopic Imaging,” PNAS Nexus 4, no. 3 (2025): pgaf072, 10.1093/pnasnexus/pgaf072. 40109558 PMC11922071

