# Supplementary figures and images for: Validation of Dynamic Deuterium Metabolic Imaging (DMI) for the Measurement of Cerebral Metabolic Rates of Glucose in Rat
Source: NMR Biomed. 2025 Dec 10;39(1):e70194. doi: 10.1002/nbm.70194 (PMC12695439; doi:10.1002/nbm.70194)

# POCE-difference $^1\text{H}$ - $^{13}\text{C}$ -MRS

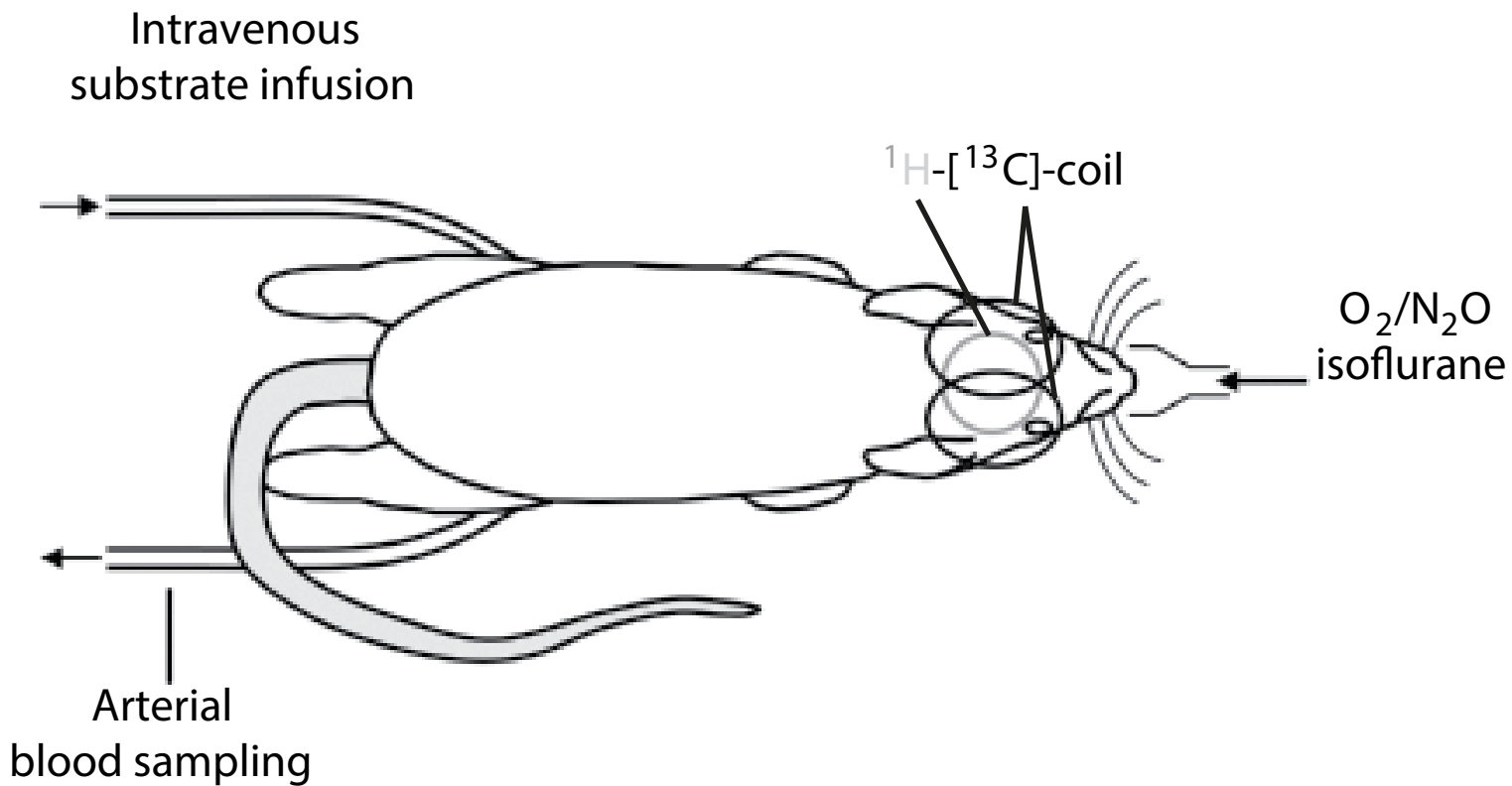

## $^2\text{H}$ -MRS

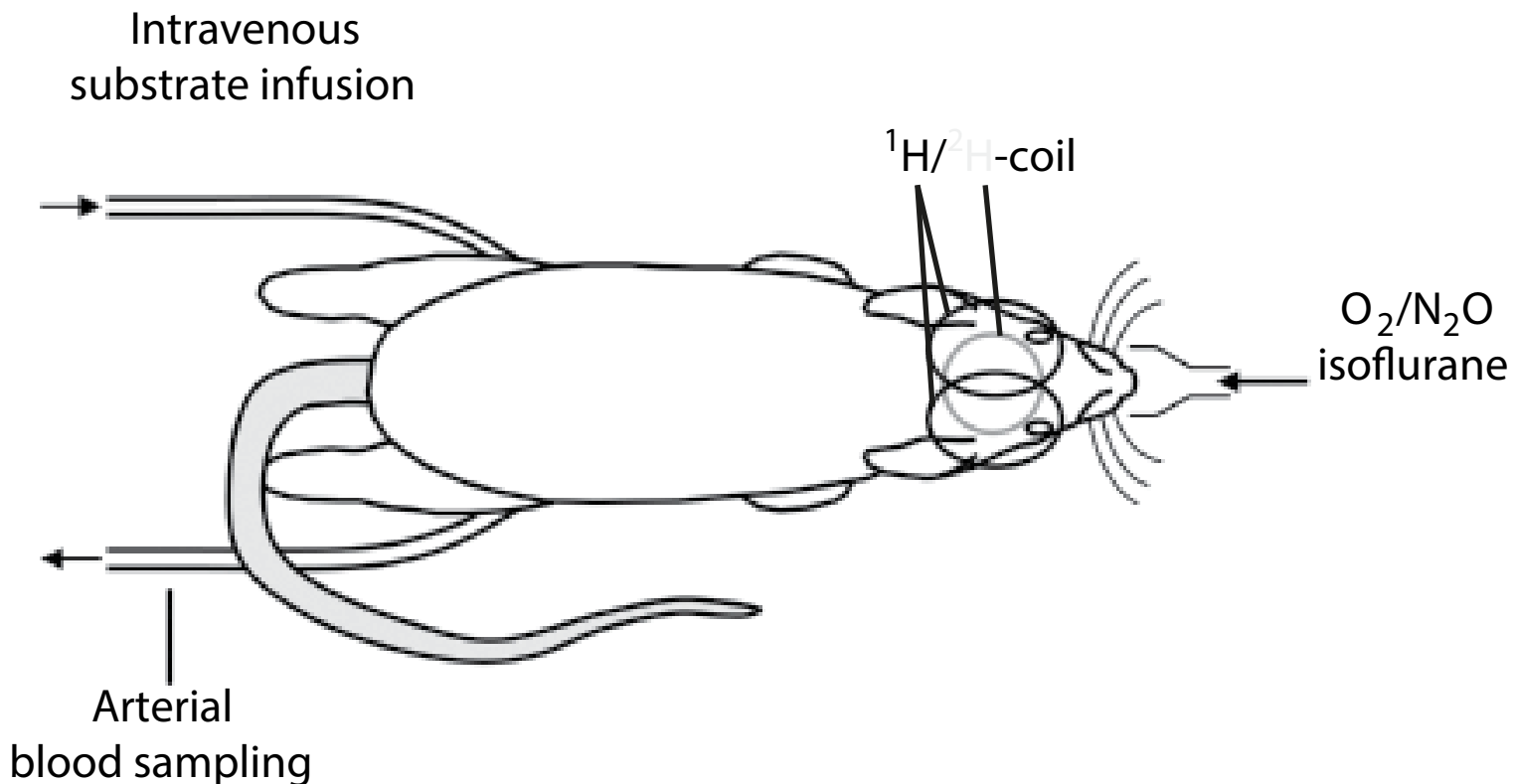

Supplement: Supplementary file 1 — Figure S1: Coil configuration and positioning for 1H‐[13C] MRS (top) and 2H MRS (bottom). [file NBM-39-e70194-s001.pdf]

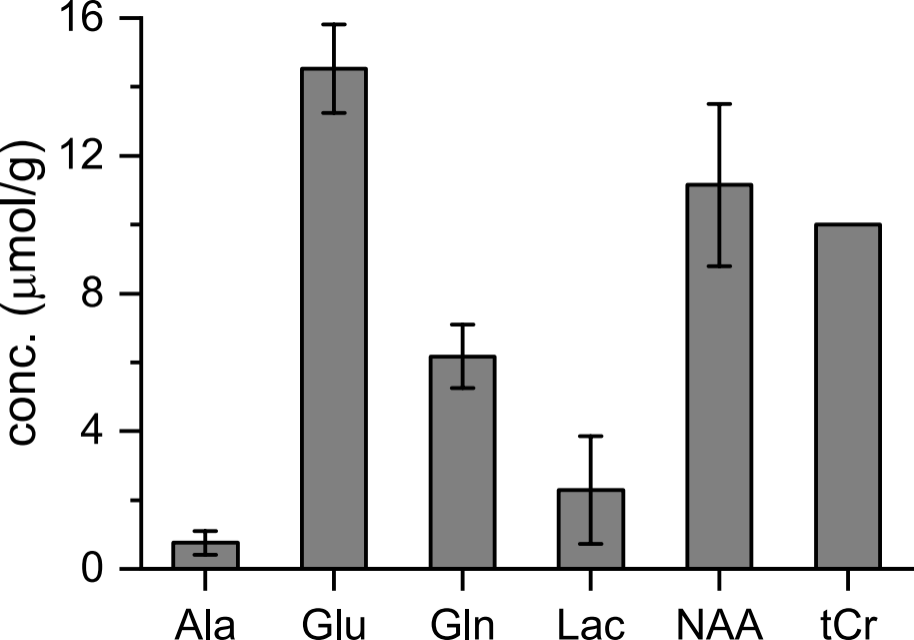

Supplement: Supplementary file 2 — Figure S2: Total metabolic concentrations (pool sizes) as obtained from in vivo total 1H‐[13C] MRS under the assumption of a 10 μmol/g tCr concentration. Total Lac concentration obtained from metabolic modeling of 1H‐[13C] MRS data. [file NBM-39-e70194-s004.pdf]
