# Supplementary material for: Validation of Dynamic Deuterium Metabolic Imaging (DMI) for the Measurement of Cerebral Metabolic Rates of Glucose in Rat
Source: NMR Biomed. 2025 Dec 10;39(1):e70194. doi: 10.1002/nbm.70194 (PMC12695439; doi:10.1002/nbm.70194)
Supplement: Supplementary file 4 — Data S1: Supplementary equation. [file NBM-39-e70194-s002.docx]

**Equations**

**Mass balance, same for ^2^H MRS and ^1^H-[^13^C] MRS**

$$\frac{d{[Glc}_{i}(t)]}{dt}=\frac{V_{max,glc}[{Glc}_{p}\left( t \right)]}{K_{M,glc}+[{Glc}_{p}\left( t \right)]}-\frac{V_{max,glc}{[Glc}_{i}\left( t \right)]}{K_{M,glc}V_{d}+{[Glc}_{i}\left( t \right)]}-{CMR}_{gl}$$

with K_M,glc_ = 13.9 mM, V_max,glc_ = 5.8xCMR_gl_ (Mason et al., 1992) as starting values for iteration (limits ±75% of starting value) and volume fraction of glucose (V_d_ = 0.78 mL/g, Buschiazzo et al., 1970)

$$\frac{d[Lac]}{dt}=2{CMR}_{gl}+V_{lac,in}+V_{dil,gly}-V_{pdh}-V_{lac,out}-V_{lac,out,brain}=0$$

with $V_{lac,in}=\frac{V_{max,lac}[{Lac}_{p}\left( t \right)]}{K_{M,lac}+[{Lac}_{p}\left( t \right)]}+K_{D,lac}\left[ {Lac}_{p}\left( t \right) \right]=V_{lac,out}$ and V_dil,gly_ ≈ V_lac,out,brain_

$$\frac{d[Glu]}{dt}=V_{pdh}+V_{dil,tca}+V_{gln}-V_{tca}-V_{gln}=0$$

with $V_{gln}=-0.1 \mu mol/min/g+V_{tca}/2$ as Glu and Gln are treated as one combined Glx pool within the model.

$$\frac{d[Gln]}{dt}=V_{gln}-V_{gln}=0$$

**Isotope balance for ^2^H MRS / changes to ^1^H-[^13^C] MRS below marked in red**

$$\frac{d{[Glc}_{i}(t){6,6}^{*}]}{dt}=\frac{V_{max,glc}[{Glc}_{p}\left( t \right)]}{K_{M,glc}+[{Glc}_{p}\left( t \right)]}\frac{{[Glc}_{p}{6,6}^{*}]}{{[Glc}_{p}]}-\left( \frac{V_{max,glc}{[Glc}_{i}\left( t \right)]}{K_{M,glc}V_{d}+{[Glc}_{i}\left( t \right)]}-{CMR}_{gl} \right)\frac{{[Glc}_{i}{6,6}^{*}]}{{[Glc}_{i}]}$$

$$\frac{d\left[ Lac{3,3}^{*} \right]}{dt}=2*0.5*\boldsymbol{0.843}*{CMR}_{gl}\frac{{[Glc}_{i}{6,6}^{*}]}{{[Glc}_{i}]}+V_{lac,in}\frac{{[Lac}_{p}{3,3}^{*}]}{{[Lac}_{p}]}+\frac{[{NA}^{*}]}{[NA]}*V_{dil,gly}-{(V}_{pdh}+V_{lac,out}+V_{lac,out,brain})\frac{[Lac{3,3}^{*}]}{[Lac]}$$

where [NA*]/[NA] indicates the percentage of label by natural abundance, 0.0115% for ^2^H MRS and 1.1% for ^1^H-[^13^C] MRS. Red labeled isotope fraction denotes ^2^H label loss of 15.7% (de Graaf et al., 2020).

$$\frac{d[Glu{4,4}^{*}]}{dt}=V_{pdh}*\boldsymbol{0.615}*\frac{[Lac{3,3}^{*}]}{[Lac]}+\frac{[{NA}^{*}]}{[NA]}*V_{dil,tca}+V_{gln}\frac{[Gln{4,4}^{*}]}{[Gln]}-(V_{tca}+V_{gln})\frac{[Glu{4,4}^{*}]}{[Glu]}$$

$$\frac{d[Gln{4,4}^{*}]}{dt}=V_{gln}\frac{[Glu{4,4}^{*}]}{[Glu]}-V_{gln}\frac{[Gln{4,4}^{*}]}{[Gln]}$$

Red labeled isotope fraction denotes ^2^H label loss of the concentration and FE (both measured by ^1^H-[^13^C] MRS) weighted average of previously reported label loss of 37.9% for Glu and 41.5% of Gln, resulting in 38.5% label loss of Glx (de Graaf et al., 2020).

**Isotope balance for ^1^H-[^13^C] MRS**

$$\frac{d{[Glc}_{i}(t)1^{*}]}{dt}=\frac{V_{max,glc}[{Glc}_{p}\left( t \right)]}{K_{M,glc}+[{Glc}_{p}\left( t \right)]}\frac{{[Glc}_{p}1^{*}]}{{[Glc}_{p}]}-\left( \frac{V_{max,glc}{[Glc}_{i}\left( t \right)]}{K_{M,glc}V_{d}+{[Glc}_{i}\left( t \right)]}-{CMR}_{gl} \right)\frac{{[Glc}_{i}1^{*}]}{{[Glc}_{i}]}$$

$$\frac{d\left[ Lac3^{*} \right]}{dt}=2*0.5*{CMR}_{gl}\frac{{[Glc}_{i}1^{*}]}{{[Glc}_{i}]}+V_{lac,in}\frac{{[Lac}_{p}3^{*}]}{{[Lac}_{p}]}+\frac{[{NA}^{*}]}{[NA]}*V_{dil,gly}-{(V}_{pdh}+V_{lac,out}+V_{lac,out,brain})\frac{[Lac3^{*}]}{[Lac]}$$

where [NA*]/[NA] indicates the percentage of label by natural abundance, 1.1% for ^1^H-[^13^C] MRS

$$\frac{d[Glu4^{*}]}{dt}=V_{pdh}\frac{[Lac3^{*}]}{[Lac]}+\frac{[{NA}^{*}]}{[NA]}*V_{dil,tca}+V_{gln}\frac{[Gln4^{*}]}{[Gln]}-(V_{tca}+V_{gln})\frac{[Glu4^{*}]}{[Glu]}$$

$$\frac{d[Gln4^{*}]}{dt}=V_{gln}\frac{[Glu4^{*}]}{[Glu]}-V_{gln}\frac{[Gln4^{*}]}{[Gln]}$$
