# Supplementary material for: Validation of Dynamic Deuterium Metabolic Imaging (DMI) for the Measurement of Cerebral Metabolic Rates of Glucose in Rat
Source: NMR Biomed. 2025 Dec 10;39(1):e70194. doi: 10.1002/nbm.70194 (PMC12695439; doi:10.1002/nbm.70194)
Supplement: Supplementary file 5 — Data S2: Supplementary information. [file NBM-39-e70194-s005.docx]

Table S1:

| 1. Hardware | ^2^H MRS | ^1^H-[^13^C] MRS |
| --- | --- | --- |
| a. Field strength [T] | 11.7 T | 11.7 T |
| b. Manufacturer | Magnex (magnet), Bruker (spectrometer) | Magnex (magnet), Bruker (spectrometer) |
| c. Model (software version if available) | ParaVision 6 | ParaVision 6 |
| d. RF coils: nuclei (transmit/ receive), number of channels, type, body part | ^2^H: Two-turn 20 mm x 15 mm elliptical surface coil  ^1^H: Two orthogonal 20 mm diameter coils driven in quadrature | ^13^C: Two orthogonal 20 mm diameter coils driven in quadrature  ^1^H: single-turn 14-mm diameter coil |
| e. Additional hardware | RF amplifiers for ^1^H/^2^H/^13^C transmission | RF amplifiers for ^1^H/^2^H/^13^C transmission |

Table S2:

| 2. Acquisition MRI | ^2^H MRS | ^1^H-[^13^C] MRS |
| --- | --- | --- |
| a. Pulse sequence | Gradient-echo | Gradient-echo |
| b. Volume of Interest (VOI) locations | See Fig. 2A | See Fig. 2A |
| c. Nominal VOI size [cm^3^, mm^3^] | 27.0 x 27.0 mm^2^ and 6 slices of 1.0 mm thickness | 25.5 x 25.5 mm^2^ and 6 slices of 1.0 mm thickness |
| d. Repetition Time (TR), Echo Time (TE) [ms, s] | TR 100 ms  TE 3.3 ms | TR 3000 ms  TE 2 ms |
| e. Total number of Excitations or acquisitions per spectrum  In time series for kinetic studies   1. Number of Averaged spectra (NA) per time-point 2. Averaging method (e.g. block-wise or moving average) 3. Total number of spectra (acquired / in time-series) | n.a. | n.a. |
| f. Additional sequence parameters  (spectral width in Hz, number of spectral points, frequency offsets)  If STEAM:, Mixing Time (TM)  If MRSI: 2D or 3D, FOV in all directions, matrix size, acceleration factors, sampling method | FA 30° | FA 45° |
| g. Water Suppression Method | n.a. | n.a. |
| h. Shimming Method, reference peak, and thresholds for “acceptance of shim” chosen | n.a. | n.a. |
| i. Triggering or motion correction method  (respiratory, peripheral, cardiac triggering, incl. device used and delays) | n.a. | n.a. |

| 2. Acquisition MRS | ^2^H MRS | ^1^H-[^13^C] MRS |
| --- | --- | --- |
| a. Pulse sequence | Spin-echo | Proton-observed carbon-edited (POCE) |
| b. Volume of Interest (VOI) locations | See Fig. 2A | See Fig. 2A |
| c. Nominal VOI size [cm^3^, mm^3^] | 6x3x6 mm³ | 6x1.5x6 mm³ (before spectral quantification combination of 2 slices to 6x3x6 mm³) |
| d. Repetition Time (TR), Echo Time (TE) [ms, s] | TR 800 ms  TE 8 ms | TR 4000 ms  TE 21 + 7.8 ms |
| e. Total number of Excitations or acquisitions per spectrum  In time series for kinetic studies   1. Number of Averaged spectra (NA) per time-point 2. Averaging method (e.g. block-wise or moving average) 3. Total number of spectra (acquired / in time-series) | NA 64 per time-point, block-wise averaging, total scan time about 120 min | NA 4 per time-point, block-wise averaging, total scan time about 120 min |
| f. Additional sequence parameters  (spectral width in Hz, number of spectral points, frequency offsets)  If STEAM:, Mixing Time (TM)  If MRSI: 2D or 3D, FOV in all directions, matrix size, acceleration factors, sampling method | Spectral width 5 kHz, 9 phase encoding steps, 1980 spectral points | Spectral width 10 kHz, 17 phase encoding steps, 1980 spectral points; for macromolecular baseline TR = 5000 ms, TI_1_ = 1950 ms, TI_2_ = 550ms |
| g. Water Suppression Method | n.a. | VAPOR |
| h. Shimming Method, reference peak, and thresholds for “acceptance of shim” chosen | Second-order spherical harmonical shimming after B_0_-mapping. Water linewidth <30 Hz in 8x5x8 mm³ | Second-order spherical harmonical shimming after B_0_-mapping. Water linewidth <25 Hz in 7x4x7 mm³ |
| i. Triggering or motion correction method  (respiratory, peripheral, cardiac triggering, incl. device used and delays) | n.a. | n.a. |

| 3. Data analysis methods and outputs | ^2^H MRS | ^1^H-[^13^C] MRS |
| --- | --- | --- |
| a. Analysis software | MATLAB, NMRWizard, CWave, home-written scripts | MATLAB, NMRWizard, CWave, home-written scripts |
| b. Processing steps deviating from quoted reference or product | Described in method sections 2.4 and 2.5 | Described in method sections 2.4 and 2.5 |
| c. Output measure  (e.g. absolute concentration, institutional units, ratio)Processing steps deviating from quoted reference or product | Flux rates of TCA cycle V_tca_ and glucose consumption CMR_gl_ | Flux rates of TCA cycle V_tca_ and glucose consumption CMR_gl_ |
| d. Quantification references and assumptions, fitting model assumptions | Metabolite concentrations based on pre-infusion, natural abundance water signal, equal to 10.12 mM; assumptions for metabolic modeling described in 2.5 | Total metabolite concentrations based on total creatine as internal standard to 10 µmol/g; assumptions for metabolic modeling described in 2.5 |

| 4. Data Quality | ^2^H MRS | ^1^H-[^13^C] MRS |
| --- | --- | --- |
| a. Reported variables  (SNR, Linewidth (with reference peaks)) | *Not reported* | *Not reported* |
| b. Data exclusion criteria | *None excluded*  *Maximum CRLBs of last time point*  *Glucose 5 %*  *Glx 5 %*  *Lactate 25 %*  *Water 2 %* | *Two excluded*  *Maximum CRLBs of last time point*  *GluH4 5 %*  *GlnH4 25 %*  *LacH3 15 %*  *Physiologically unexplainable signal losses of fitted GluH4 signal of ≥25% compared to previous data point in one time series* |
| c. Quality measures of postprocessing Model fitting (e.g. CRLB, goodness of fit, SD of residual) | *CRLBs (mean±standard deviation of last time point):*  *Glucose 3.6±0.7 %*  *Glx 3.7±0.2 %*  *Lactate 11.8±4.5 %*  *Water 1.0±0.1 %* | *CRLBs (mean±standard deviation of last time point):*  *GluH4 1.7±0.5 %*  *GlnH4 9.9±5.9 %*  *LacH3 5.2±2.3 %* |
| d. Sample Spectrum | Fig. 2 B; dynamics Fig. 3 | Fig. 2 C/D, dynamics Fig. 4 |
